# Disruption of Sarcoendoplasmic Reticulum Calcium ATPase Function in *Drosophila* Leads to Cardiac Dysfunction

**DOI:** 10.1371/journal.pone.0077785

**Published:** 2013-10-03

**Authors:** Dennis M. Abraham, Matthew J. Wolf

**Affiliations:** Department of Medicine, Duke University Medical Center, Durham, North Carolina, United States of America; University of Massachusetts Medical School, United States of America

## Abstract

Abnormal sarcoendoplasmic reticulum Calcium ATPase (SERCA) function has been associated with poor cardiac function in humans. While modifiers of SERCA function have been identified and studied using animal models, further investigation has been limited by the absence of a model system that is amenable to large-scale genetic screens. *Drosophila melanogaster* is an ideal model system for the investigation of SERCA function due to the significant homology to human SERCA and the availability of versatile genetic screening tools. To further the use of *Drosophila* as a model for examining the role of SERCA in cardiac function, we examined cardiac function in adult flies. Using optical coherence tomography (OCT) imaging in awake, adult *Drosophila*, we have been able to characterize cardiac chamber dimensions in flies with disrupted in *Drosophila* SERCA (CaP60A). We found that the best studied CaP60A mutant, the conditional paralytic mutant CaP60A^kum170^, develops marked bradycardia and chamber enlargement that is closely linked to the onset of paralysis and dependent on extra cardiac CaP60A. In contrast to prior work, we show that disruption of CaP60A in a cardiac specific manner results in cardiac dilation and dysfunction rather than alteration in heart rate. In addition, the co-expression of a calcium release channel mutation with CaP60A ^kum170^ is sufficient to rescue the cardiac phenotype but not paralysis. Finally, we show that CaP60A overexpression is able to rescue cardiac function in a model of *Drosophila* cardiac dysfunction similar to what is observed in mammals. Thus, we present a cardiac phenotype associated with *Drosophila* SERCA dysfunction that would serve as additional phenotyping for further large-scale genetic screens for novel modifiers of SERCA function.

## Introduction

Derangements in calcium handling have been implicated as common pathway in cardiac dysfunction. A major regulator of myocyte calcium homeostasis is the sarcoendoplasmic reticulum ATPase pump (SERCA), which is responsible for removing a significant fraction of calcium from the cytosol into the sarcoplasmic reticulum after cardiac contraction [1]. Each cardiac contraction represents the coordinated action of calcium into and removal out of the cytosol. Perturbations in this finely coordinated mechanism, such as in altered SERCA function, can lead to cell and, ultimately, organ dysfunction over time [2]. Regulators of SERCA function in mammals, such as phospholamban [3] and sarcolipin [4], have been examined as potential therapeutic targets. In preclinical models of and in humans suffering from dilated cardiomyopathy, manipulation of SERCA function, through overexpression of SERCA or the manipulation of known modifiers, has been shown to improve cardiac function [5,6]. Whether additional modifiers of SERCA function exist in mammals is unknown.

The search for novel modifiers of SERCA in mammalian model systems is limited by cost, complexity and the lack of available genetic tools for screening. However, *Drosophila melanogaster* is a model system that has robust genetic tools and has been uniquely adapted to perform large-scale genetic screens [7]. In addition, publicly available *Drosophila* stocks, including those bearing mutant alleles, transgenic RNAi, molecularly defined deletions and insertional disruptions of genes, present a large arsenal of tools to investigate the molecular underpinnings of gene function. *Drosophila* express one thapsigargin sensitive Calcium ATPase channel (called Calcium ATPase at 60A or *CaP60A*) [8], whereas vertebrates have multiple isoforms of SERCA [9]. The high degree of conservation between *Drosophila* SERCA and human SERCA make it an ideal model system for studying its function *in vivo* [10]. Interestingly, while a number of functional domains are conserved (ATP and thapsigargin binding site) the phospholamban binding site is not, suggesting that *Drosophila* may modulate SERCA function in a manner which may be different but potentially relevant to mammals [11]. In fact, a novel gene called *sarcolamban* has been recently identified that encodes small bioactive peptides and modulates *Drosophila Ca-P60A* [12]. Additionally, alterations in *Drosophila* SERCA function have been shown to have profound effects on skeletal muscle function [13] and affect larval heart rate [11], although its role in adult cardiac function is not well understood.

A limitation in the study of SERCA function in the *Drosophila* heart has been the ability to assess and accurately quantify cardiac function in the adult fly. Previously, we described a technique to non-invasively assess cardiac function in awake, adult flies using optical coherence tomography (OCT) [14]. This technique provides detailed functional data of the adult fly heart in a manner similar to echocardiography in humans and has been exploited for screening of genetic modifiers with relevance to cardiac function [15–19]. Using OCT, we assessed cardiac function in publicly available models of SERCA loss of function, including the paralytic mutant CaP60A^kum170^, and gain of function using cardiac specific overexpression of SERCA. To test whether modifiers of SERCA function could be identified using OCT; we investigated the effect of co-expressing a calcium release channel mutation on the *CaP60A* ^*kum170*^ phenotype. Finally, we tested whether SERCA overexpression can augment cardiac function in a manner similar to that observed in mammals, using a model of *Drosophila* cardiac dysfunction. Thus, we show that SERCA dysfunction leads to abnormal cardiac function in the adult fly, manifested as both alterations in chamber dimension and in rhythmicity, that the manipulation of other non-SERCA elements of calcium signaling can modify these phenotypes and the SERCA overexpression can rescue cardiac function. Taken together, these data show that disruption of *Drosophila* SERCA results in a cardiac phenotype that shares some similarities to mammals and, depending on the screening approach, *Drosophila melanogaster* can be used as a powerful model system to identify novel modifiers of SERCA.

## Material and Methods

### *Drosophila* Stocks

*CaP60A* ^*kum170*^, *UAS- CaP60A RNAi*, *ITP-r83A* ^*MB03611*^, *Df*(2R) *BSC60*, *CaP60A* ^KG00570^ and *Rya-44F* ^16^ stocks were obtained from the Bloomington *Drosophila* Stock Center. All stocks were maintained on standard yeast protein media at room temperature. The *tinC-Gal4* stock was kindly provided by Manfred Frasch [20]. The TARGET system was used based on pre-existing stocks to generate *p*{*tubulin-Gal80* ^*TS*^}*; p*{*tinC-Gal4*} stocks based on previously described methods [21].

### Generation of CaP60A Transgenic Stocks

The cDNA encoding wild type *CaP60A* and *CaP60A* ^*kum170*^ were isolated by RT-PCR from *w* ^1118^ and *CaP60A* ^kum170^ flies, subcloned into pCasper5 and verified by sequencing. Transgenic *Drosophila* harboring either the wild type *CaP60A* or *CaP60A* ^*kum170*^ were generated by established methods [20].

### Post Developmental Expression of siRNA

Flies bearing *UAS-RNAi* to *CaP60A* were bred with *tubulin-Gal80* ^*TS*^ *; tinC-Gal4* stocks, creating a fly bearing these transgenes. Flies were bred at 18°C until eclosion, at which time half were moved to 27°C. At 27°C, Gal80 repression of Gal4 is released and siRNA to CaP60A is expressed. Flies were kept at either 18°C (control condition) or 27°C (transgene is on) for 7 days, after which time cardiac function was assessed.

### Cardiac Measurements using OCT

Cardiac function in adult *Drosophila* was measured using a custom built OCT microscopy system (Bioptigen, Inc, Durham, NC) as previously described [14].

Briefly, adult female *Drosophila* between 7 and 10 days post eclosion were briefly subjected to CO_2_, placed on a soft gel support, and allowed to fully awaken based on body movement. OCT M-modes were recorded and images were processed using ImageJ software using a 125µm standard. End-diastolic (EDD) and end-systolic (ESD) were determined from three consecutive heartbeats. Heart rate was determined by counting the total number of beats which occurred during a 2.6 second or 5.2 second recording and calculating the number of beats per minute (bpm). Fractional shortening (FS) was calculated as [EDD-ESD]/EDD x 100. Fractional shortening is a calculation of the percentage change in cardiac chamber dimensions during contraction and a decrease in fractional shortening is interpreted as a reduction in systolic function.

Induction of paralysis by heat shock was accomplished in the following manner: awake flies were transferred to a glass vial using a funnel and without anesthesia, glass vials were then immersed in a water bath heated to 40°C for 10 minutes and then moved to a clean plastic vial for 1 hour prior to assessment of cardiac function. To minimize the potential effect of CO_2_ on heart rate in the paralytic mutants, paralyzed flies were embedded in the gel support without the use of CO_2_.

### Statistical Analysis

Comparisons of chamber dimensions or heart rates were determined by an analysis of variances (ANOVA) with Tukey’s test for multiple comparisons when necessary. GraphPad Prism statistical software (GraphPad Software Inc.) was used for all analyses.

## Results

**Figure 1:**
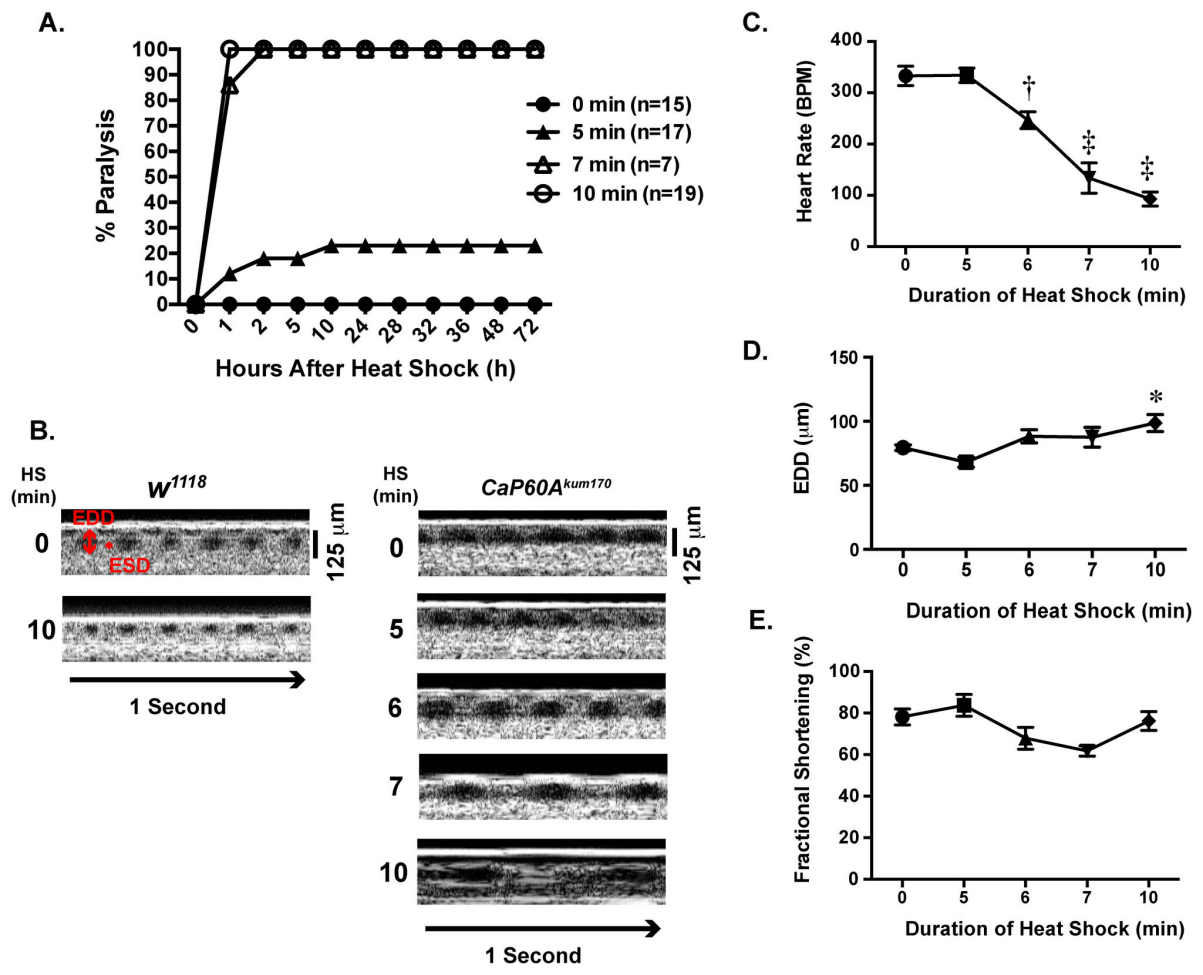
*CaP60A* ^*kum170*^ mutant has altered heart rate and cardiac dimensions after heat shock. **A**. Percent paralysis after heat shock. *CaP60A* ^kum170^ heterozygote flies were exposed to heat shock of varying durations (no heat shock defined as 0 min (closed circle), 5 min (closed triangles), 7 min (open triangles), or 10 min (open circles)) and observed for up to 72 hours. All flies receiving a heat shock of greater than 7 minutes developed irreversible paralysis; while a smaller percentage of flies developed paralysis after 5-minute heat shock. **B**. Representative optical coherence tomography (OCT) recordings from *w* ^1118^ and *CaP60A* ^kum170^ after varying durations of heat shock (HS). End diastolic dimension (EDD) and end systolic dimension (ESD) are denoted in red; A 125 micron standard and one-second bar are shown. **C**. Heart rates measured from 3 second OCT recordings show a progressive decline in heart rate with increasing durations of heat shock. **D**. End diastolic dimensions (EDDs) increase with increasing duration of heat shock. **E**. Fractional shortening is not markedly altered with heat shock in the CaP60A^kum170^ mutants. *p<0.05, † p<0.005, ‡p<0.0001 in comparison to 0 minutes heat shock by one-way ANOVA with Tukey’s multiple comparisons test. N= 16, 12, 16, 4, 22 for 0, 5, 6, 7 and 10 minute groups, respectively.

Previous work suggested that SERCA primarily maintains heart rate in *Drosophila* [11,13], a finding that differs from the observed function of SERCA in the mammalian heart to maintain normal contractile function [22] and may be due to limitations of the techniques to measure adult *Drosophila* cardiac function. On the basis of previous findings, we tested whether global disruption of SERCA in adult flies would affect heart function due to dysregulation of cardiac calcium handling. To address this, we first examined the cardiac function of paralytic mutant *CaP60A* ^*kum170*^ using optical coherence tomography (OCT). The *CaP60A* ^*kum170*^ mutant was isolated from an ethyl methanesulfonate genetic screen designed to identify temperature-sensitive paralytic mutants [13]. *CaP60A* ^*kum170*^ flies bear a point mutation in *Drosophila* SERCA, which is an amino acid replacement of glutamic acid for lysine at position 442 (E442K). The E442K mutation is located in the hinge domain of SERCA and has been proposed to potentially influence either ATP binding or conformational state of the molecule [13]. For our studies, we also used *w* ^1118^, a common laboratory stock, as a control. The cardiac and paralysis phenotype of the *CaP60A* ^*kum170*^ flies were induced by exposure to heat shock at 40°C for 10 minutes. We examined the effect of varying duration of heat shock on both paralysis and cardiac function. All *CaP60A* ^*kum170*^ subjected to a 7 or 10-minute duration of heat shock developed paralysis, while 20% of flies receiving a 5-minute heat shock developed paralysis (Figure 1A). *CaP60A* ^*kum170*^ flies that developed paralysis did not recover normal function for up to 72 hours after heat shock (Figure 1A). Heart rate measurements were made using 2.6 second or 5.6 seconds recordings, which were then used to calculate the number of heart beats per minute. OCT images revealed an inverse relationship between heart rate and the duration of heat shock with a 72% reduction in heart rate after 10-minute heat shock (Figure 1B & 1C), which was coupled with 19% increase in end diastolic dimension (EDD). However, there was no significant change in fractional shortening, a surrogate for cardiac function, with increasing duration of heat shock. In comparison, *w* ^1118^ flies did not develop paralysis or significant changes in cardiac function or heart rate.

**Figure 2:**
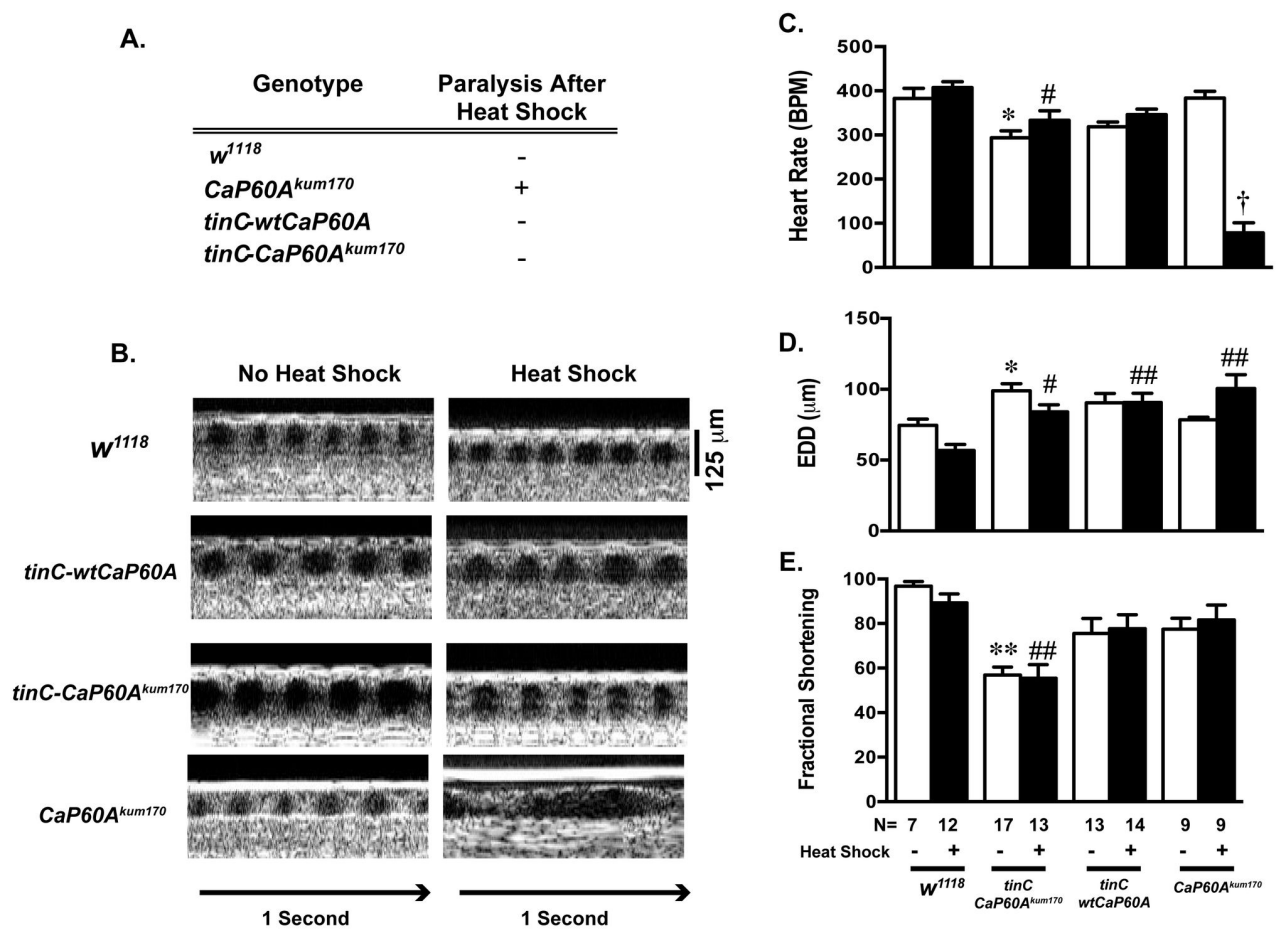
The effects of cardiac specific overexpression of *CaP60A* ^kum170^ and *wild type CaP60A* on cardiac phenotypes. **A**. Cardiac specific overexpression of wild type CaP60A (*tinC–wtCaP60A*) or mutant CaP60A^kum170^ (*tinC-CaP60A^kum170^*) in *Drosophila* is not sufficient to phenocopy the paralysis phenotype of the global *CaP60A* ^*kum170*^ mutant. Each mutant had two copies of the transgene; **B**. Representative OCT images from transgenic CaP60A overexpression flies in comparison to *w* ^1118^ and global *CaP60A* ^*kum170*^. 125 micron standard and one-second bars are shown. **C**. Heart rate, D. End diastolic dimensions (EDD), **E**. Fractional shortening (%) in *w* ^1118^, *tinC–wtCaP60A* and *tinC-CaP60A* ^*kum170*^ and *CaP60A* ^*kum170*^ at baseline and after 10-minute heat shock. No significant differences were noted in the heart rate in either *tinC–wtCaP60A* or *tinC-CaP60A* ^*kum170*^ at baseline and after heat shock compared to *w* ^1118^. *p<0.05, **p<0.005 vs. *w* ^1118^ no heat shock, #p<0.05, # # p<0.005 vs. *w* ^1118^ with heat shock, † p<0.0001 vs. *w* ^1118^ with heat shock, *tinC-CaP60A* ^*kum170*^ with heat shock, *tinC-CaP60A* ^kum170^ with heat shock and *CaP60A* ^*kum170*^ no heat shock by one-way ANOVA with Tukey’s multiple comparisons test.

**Figure 3:**
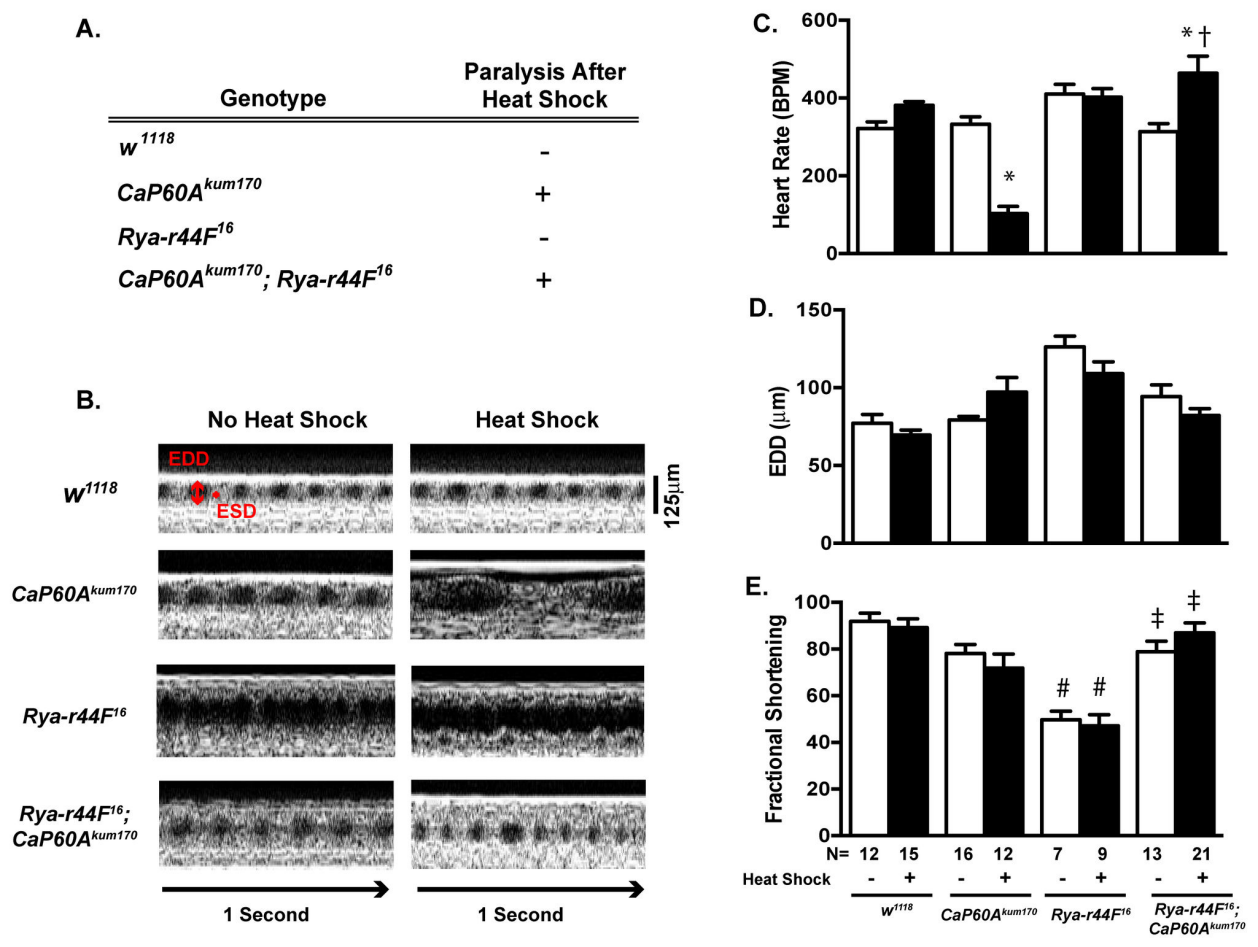
The effects of Calcium Release mutations on the *CaP60A* ^kum170^ cardiac phenotypes. **A**. Heat shock induced paralysis in heterozygous *CaP60A* ^*kum170*^ flies as well as in transheterozygous mutants carrying both *CaP60A* ^*kum170*^ and a mutation in Ryanodine receptor (*Rya-r44F^16^*). No heat shock induced paralysis was noted in the single *Rya-r44F* ^16^ mutants or *w* ^1118^. **B**. Representative OCT images from *w* ^1118^, *CaP60A* ^*kum170*^, *Rya-r44F* ^16^, *CaP60A* ^*kum170*^; *Rya-r44F* ^16^ with and without 10-minute heat shock; A 125 micron standard and one-second bar are shown. EDD= End diastolic dimension, ESD=End systolic dimension. **C**. Heart rate, D. EDD and **E**. Fractional Shortening (%) in *w* ^1118^, *CaP60A* ^*kum170*^, *Rya-r44F* ^16^, *CaP60A* ^*kum170*^ *; Rya-r44F* ^16^ at baseline and after 10-minute heat shock. *p<0.0002 vs. non-heat shock state of the same genotype, † p<0.0001 vs. *CaP60A* ^*kum170*^ after heat shock, #p<0.0001 vs. *w* ^1118^ of the same treatment condition, ‡p<0.0002 vs. *Rya-r44F* ^16^ of the same treatment condition by one-way ANOVA with Tukey’s multiple comparisons test.

In order to investigate whether the changes in heart rate in the *CaP60A* ^*kum170*^ were due to intrinsic cardiac process, we generated transgenic *Drosophila* that specifically overexpressed either wild type SERCA (tinC*-wtCaP60A*) or the *CaP60A* ^*kum170*^ mutant SERCA (*tinC-CaP60A* ^*kum170*^) in cardiac tissue. Cardiac specific overexpression of wild type or mutant SERCA was not sufficient to induce paralysis after heat shock (Figure 2A). However, overexpression of *CaP60A* ^*kum170*^ resulted in both cardiac dilation (Figure 2B, 2D) and diminished cardiac function (Figure 2B, 2E) that was independent of exposure to 10 minutes of heat shock compared to *w* ^1118^. No significant decrement in cardiac function was noted in the presence of overexpression of wild type SERCA in comparison to *w* ^1118^. Unlike the flies that globally express *CaP60A* ^*kum170*^, flies with cardiac specific overexpression *CaP60A* ^*kum170*^ did not have a decreased heart rate with heat shock (Figure 2C). Next, we sought to determine whether the altering calcium release in *Drosophila* would affect the cardiac function of *CaP60A* ^*kum170*^ flies using a genetic approach. To accomplish these we introduced a mutation in Ryanodine (*Rya-r44F* ^*16*^) into the *CaP60A* ^*kum170*^ genetic background and assessed the effect on both paralysis and cardiac function. *Rya-r44F* ^16^ encodes a mutation in the Ryanodine receptor resulting in a deletion that extends from the *p*{*lacW*}*Rya-r44F* ^*k04913*^ insertion to include the first coding exon and the first and second introns resulting in a hypomorphic allele [23]. The *Rya-r44F* ^16^ mutant exhibits diminished cardiac function that is independent of heat shock (Figure 3B & 3E), but does not exhibit any heat shock induced paralysis (Figure 3A) or change in heart rate (Figure 3C). Addition of *Rya-r44F* ^16^ in the context of *CaP60A* ^kum170^ (*Rya-r44F* ^*16*^ *; CaP60A* ^*kum170*^) did not result in any change in the paralysis phenotype of the *CaP60A* ^kum170^ (Figure 3A). However, the addition of *Rya-r44F* ^16^ rescued the heart rate phenotype *CaP60A* ^kum170^ mutant (Figure 3C) and improved cardiac dysfunction phenotype of the *Rya-r44F* ^16^ mutant (Figure 3E). No significant changes in end diastolic dimensions or fractional shortening were noted in the *Rya-r44F* ^*16*^ *; CaP60A* ^*kum170*^ at baseline or after heat shock. These data suggest that the heart rate phenotype observed in the CaP60A^kum170^ mutant flies result from extracardiac effects of SERCA and can be effectively uncoupled from the paralysis phenotype through introduction of sarcoplasmic reticulum calcium release mutation.

**Figure 4:**
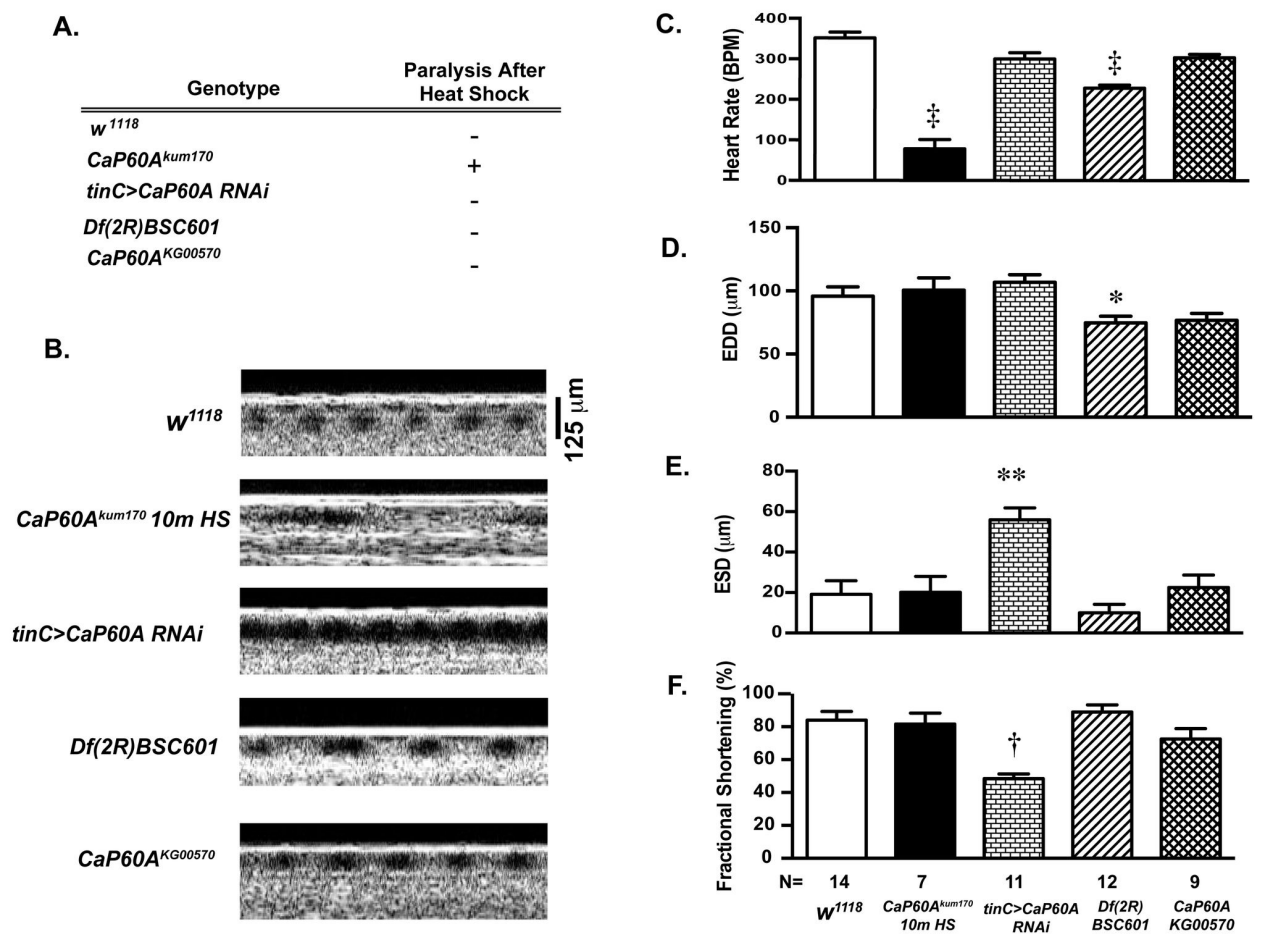
The effects of loss of CaP60A function on cardiac parameters. **A**. Heat shock did not induce paralysis in flies with cardiac specific CaP60A RNAi (*tinC>CaP60A RNAi*), heterozygous deletion of CaP60A (*Df*(2R) *BSC601*), heterozygous p-element disruption of CaP60A (*CaP60A^KG00570^*), or *w* ^1118^ compared to *CaP60A* ^*kum170*^. **B**. Representative OCT images in *w* ^1118^, *CaP60A* ^kum170^ after 10 minute heat shock, *tinC>CaP60A* *RNAi*, *Df* (2R) *BSC601* and *CaP60A* ^*KG00570*^; A 125 micron standard and one second bar are shown, C. Heart rate, D. End Diastolic Dimensions (EDD), E. End Systolic Dimensions (ESD) and **F**. Fractional shortening (%) in *w* ^*1118*^, *CaP60A* ^kum170^ after 10 minute heat shock, *tinC>CaP60A* *RNAi*, *Df*(2R) *BSC601* and *CaP60A* ^*KG00570*^. †p<0.001 vs. *w* ^1118^, *p<0.05 vs. *tinC>CaP60A* *RNAi*, ‡p<0.0001 vs. *w* ^1118^, **p<0.005 vs. *w* ^1118^ by one-way ANOVA with Tukey’s multiple comparisons test.

Although it remains unclear how global SERCA modulation affects heart rate, these data indicated that modulation of cardiac SERCA affected other important aspects of heart function, so we next sought to characterize alternative *Drosophila* models of SERCA dysfunction using OCT. To accomplish this, we studied the effect of cardiac specific RNAi targeting SERCA transcription (*tinC>CaP60A RNAi*), p-element disruption of SERCA (*CaP60A* ^*KG00570*^) and heterozygous genomic deficiency of SERCA (*Df*(2R) *BSC601*) on cardiac function and paralysis. Heat shock induced paralysis was only observed in the *CaP60A* ^kum170^ flies, but not in the other SERCA mutation stocks (Figure 4A) and this was accompanied by a marked decrease in heart rate (Figure 4B, 4E). In contrast to the *CaP60A* ^kum170^ phenotype, CaP60A RNAi post-developmentally driven in cardiac tissue resulted in a significantly diminished fractional shortening and increased end systolic dimension (ESD) (Figure 4B & 4E). Of note, no flies were obtained when CaP60A RNAi driven in cardiac tissue throughout development suggesting significant lethality. *Df*(2R) *BSC601* flies exhibited a slow heart rate phenotype in a non-heat shocked condition, although this is far less prominent in comparison to *CaP60A* ^*kum170*^ (Figure 4E), smaller EDDs (Figure 4D) and preserved systolic function (Figure 4C and 4F). These data suggest that the loss of SERCA function results in heart rate and chamber dimension phenotypes, which are variable amongst the tested mutants. Diminished cardiac function, which is observed in the mammalian phenotype of cardiac SERCA dysfunction [22], is present in the cardiac specific RNAi knockdown SERCA but is not seen in other mutants.

**Figure 5:**
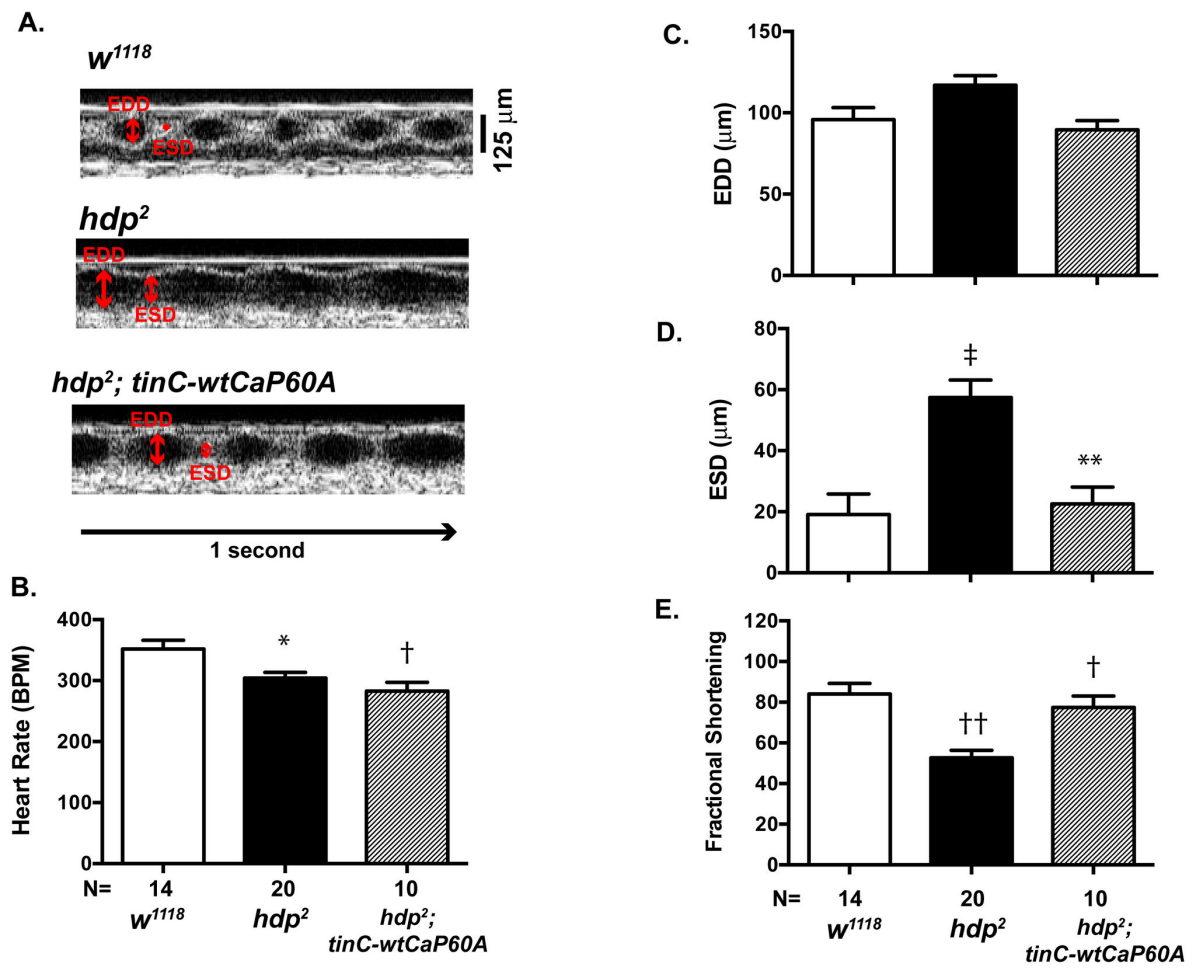
Rescue of Cardiac function with CaP60A overexpression. **A**. Representative OCT images of *w* ^1118^, *heldup* ^2^ (*hdp^2^*) and cardiac specific CaP60A (*tinC-wtCaP60A*) overexpression in *hdp* ^2^ genetic background (*hdp^2^; tinC-wtCaP60A*); Scale bar= 125µm, EDD= End diastolic dimension, ESD=End systolic dimension. **B**. Heart rate, C. EDD (µm), D. ESD (µm) and **E**. Fractional shortening (%) in *w* ^*1118*^, *CaP60A* ^kum170^ after 10 minute heat shock, *tinC>CaP60A* *RNAi*, *Df*(2R) *BSC601* and *CaP60A* ^*KG00570*^. †p<0.001 vs. *w* ^1118^, *p<0.05 vs. *tinC>CaP60A* *RNAi*, ‡p<0.0001 vs. *w* ^1118^, **p<0.005 vs. *w* ^1118^ by one-way ANOVA with Tukey’s multiple comparisons test.

The overexpression of SERCA has been shown to improve cardiac function in mammals [5,24], however it is not known whether the overexpression of CaP60A would improve cardiac function in *Drosophila*. To test whether a similar phenomenon occurs in *Drosophila*, we next investigated the effect of CaP60A overexpression on the cardiac phenotype seen in *held-up* ^2^ (*hdp* ^*2*^) mutant. Previous work revealed that the *hdp* ^2^ mutant, that are homozygous for a point mutation in troponin I, has enlarged cardiac dimension and diminished cardiac function [14]. Consistent with this work, we found that the *hdp* ^2^ mutant has significantly diminished cardiac function in comparison to *w* ^1118^ (Figure 5A, 5D & 5E). The cardiac specific overexpression of two copies of wild type CaP60A (*tinC-wtCaP60A*) in the genetic background of homozygous *hdp* ^2^ mutant (*hdp* ^2^; *tinC-wtCaP60A*) resulted in a significant improvement in cardiac function (Figure 5A, 5D & 5E). Interestingly, the heart rates of the *hdp* ^2^ mutant and *hdp* ^2^; *tinC-wtCaP60* were mildly, but significantly, diminished in comparison to *w* ^1118^ (Figure 5B). These results suggest that the overexpression of CaP60A can improve cardiac function in a model of *Drosophila* cardiomyopathy and provides further evidence that *Drosophila* can be used as a model system to study SERCA function *in vivo*.

## Discussion

Our studies reveal that the disruption of CaP60A produces altered cardiac function and rhythmicity in adult *Drosophila*. The paralytic mutant *CaP60A* ^*kum170*^ has a glutamic acid to lysine amino acid substitution at position 442 that is predicted to be located in the hinge region of CaP60A [13]. Previously, this mutation has been shown to cause a loss of CaP60A function that is associated with markedly diminished heart rates [13]. Using OCT imaging, we identified that the *CaP60A* ^*kum170*^ mutants exhibit significantly enlarged cardiac dimensions after heat shock in comparison to the basal state that is inversely related to heart rate, however systolic function remains largely unchanged. These results, coupled with our findings using cardiac specific overexpression of wild type CaP60A or *CaP60A* ^*kum170*^, suggest that the paralytic effects and cardiac phenotypes exhibited by the *CaP60A* ^*kum170*^ mutants are driven primarily by extracardiac CaP60A function in the presence of heat shock stress. Loss of function of CaP60A using RNAi (*tinC>CaP60A RNAi*), p-element disruption (*CaP60A* ^*KG00570*^) or heterozygous genomic deficiency of SERCA (*Df*(2R) *BSC601*) did not phenocopy the paralysis phenotype. Although the *Df*(2R) *BSC601* did exhibit a mild decrease in heart rate in the basal state, a diminished heart rate was not observed in either the *tinC>CaP60A RNAi* or the *CaP60A* ^*KG00570*^ animals. Strikingly, the *tinC>CaP60A RNAi* exhibits decreased fractional shortening, which is consistent with the phenotype of SERCA disruption seen in mammals [22]. Previously published work has shown that the overexpression of *CaP60A* ^*kum170*^ in muscle using a *UAS*-*CaP60A* ^*kum170*^ *with a mef2-gal4* driver resulted in a diminished heart rate, which was not seen the mutation was overexpressed in nerve tissue using *elav* ^*C155*^ *-gal4* [11]. These data, in conjunction with our findings, raise the possibility that heart rate may in part be regulated by CaP60A function in non-cardiac muscle while cardiac contraction is regulated by CaP60A in cardiac tissue.

Mutations in calcium release channels can modify the cardiac phenotype seen in the *CaP60A* ^*kum170*^ mutants, which we observed under conditions of the co-expression of the *CaP60A* ^*kum170*^ and *Rya-r44F* ^16^ mutations. However, paralysis is largely unaffected by the co-expression of *CaP60A* ^*kum170*^ and *Rya-r44F* ^16^. Our findings suggest that OCT can be used to identify potential modifiers of CaP60A activity on cardiac function, which would be have been missed by scoring of the paralysis phenotype alone.

The overexpression of SERCA is known to augment myocyte function in mammals by increasing the amplitude of calcium signals and increasing the rates of contraction and relaxation in hearts [5]. Interestingly, we observed that the overexpression of wild type CaP60A improved cardiac function in a *Drosophila* mutant that has dilated cardiomyopathy due to a mutation in troponin I (*hdp* ^*2*^). These findings significantly broaden our current understanding of how CaP60A affects cardiac function in *Drosophila melanogaster* and provide a rationale for the use of *Drosophila* as a model system to investigate mammalian SERCA function.

To date, two molecules have been identified as major regulators of calcium affinity of SERCA, phospholamban and sarcolipin. Phospholamban is a transmembrane suppressor of SERCA activity and the phosphorylation of phospholamban at Ser16 by protein kinase A (PKA) or Thr17 by calcium/calmodulin kinase II (CaMKII) results in a loss of SERCA suppression and enhanced calcium reuptake [25–27]. Genetic ablation of phospholamban results in augmented SERCA function and has been shown to augment cardiac function in muscle specific LIM protein deficient mice, a well characterized model of mammalian cardiomyopathy [3]. For example, myocytes from muscle specific LIM protein knockout mice had attenuated amplitudes of calcium transients and myocytes from the muscle specific LIM protein and phospholamban double knockout mice had the calcium transients with a shortened duration, faster decay, and preserved amplitude consistent with improved SERCA function [28]. Like phospholamban, sarcolipin is another regulator of SERCA function although is not as well understood. Sarcolipin is found primarily in the atrial tissue where it is co-expressed with and performs similar functions to phospholamban [4]. Interestingly the phospholamban binding site is not conserved in *Drosophila* CaP60A in comparison to mammalian SERCA, despite significant sequence similarity in other domains [13]. The recent discovery of *sarcolamban* represents novel insight into the regulation of SERCA [12]. Taken together, our work suggests that *Drosophila* can be used to identify additional modulators of SERCA function using OCT and would form the basis of future genetic screens to identify enhancers or suppressors.
